# Identification of MicroRNA 395a in 24-Epibrassinolide-Regulated Root Growth of *Arabidopsis thaliana* Using MicroRNA Arrays

**DOI:** 10.3390/ijms140714270

**Published:** 2013-07-09

**Authors:** Li-Ling Lin, Chia-Chi Wu, Hsuan-Cheng Huang, Huai-Ju Chen, Hsu-Liang Hsieh, Hsueh-Fen Juan

**Affiliations:** 1 Department of Life Science, National Taiwan University, Taipei 106, Taiwan; E-Mail: f94b43019@ntu.edu.tw; 2 Institute of Molecular and Cellular Biology, National Taiwan University, Taipei 106, Taiwan; E-Mail: jcwu0417@hotmail.com; 3 Institute of Biomedical Informatics, Center for Systems and Synthetic Biology, National Yang-Ming University, Taipei 112, Taiwan; 4 Institute of Plant Biology, National Taiwan University, Taipei 106, Taiwan; E-Mail: d92621103@ntu.edu.tw; 5 Graduate Institute of Biomedical Electronic and Bioinformatics, National Taiwan University, Taipei 106, Taiwan

**Keywords:** brassinosteroids, miR395a, root growth, *Arabidopsis thaliana*, microRNA array

## Abstract

Brassinosteroids (BRs) are endogenous plant hormones and are essential for normal plant growth and development. MicroRNAs (miRNAs) of *Arabidopsis thaliana* are involved in mediating cell proliferation in leaves, stress tolerance, and root development. The specifics of BR mechanisms involving miRNAs are unknown. Using customized miRNA array analysis, we identified miRNAs from *A. thaliana* ecotype Columbia (Col-0) regulated by 24-epibrassinolide (EBR, a highly active BR). We found that miR395a was significantly up-regulated by EBR treatment and validated its expression under these conditions. miR395a was over expressed in leaf veins and root tissues in EBR-treated miR395a promoter::GUS plants. We integrated bioinformatics methods and publicly available DNA microarray data to predict potential targets of miR395a. GUN5—a multifunctional protein involved in plant metabolic functions such as chlorophyll synthesis and the abscisic acid (ABA) pathway—was identified as a possible target. ABI4 and ABI5, both genes positively regulated by ABA, were down-regulated by EBR treatment. In summary, our results suggest that EBR regulates seedling development and root growth of *A. thaliana* through miR395a by suppressing GUN5 expression and its downstream signal transduction.

## 1. Introduction

In 1970, a new family of plant hormones, brassins, was reported but later found to be a mixture of multiple compounds [1]. In 1979, another steroid hormone named brassinolide was identified from rape pollen of *Brassica napus* and its structure determined [2,3]. A number of related steroid hormones have since been isolated and collectively classified under the general term brassinosteroids (BRs). To date, more than 50 BR forms including 24-epibrassinolide (EBR) have been identified in a wide variety of plant species [4]. In an attempt to understand how BRs act on plant growth and in what mechanisms they are involved, numerous studies have been and are being conducted in wide-ranging fields, including structural biology, plant physiology, molecular biology, and genetics [5]. Concurrent with biosynthetic research, a large number of BR-deficient or -insensitive mutants have been investigated, among them the *bri*1 mutant, which enabled the exploration of the affected gene’s role in BR receptor expression [6]. The components of the BRs signal transduction pathway have subsequently been studied in an effort to discriminate the relevant mechanisms [7].

BRs are structurally similar to animal and insect steroid hormones [8] and are products of the isoprenoid biosynthetic pathway; however, they differ in their subsequent metabolism of squalene-2,3-epoxide. While in animals the compound is converted to the precursor of cholesterol and steroid hormones, lanosterol, it is metabolized to cycloartenol in plants, which is the parent compound of all plant sterols [3,5,7].

In 2001, a study comparing *A. thaliana* ecotype Columbia (Col-0) with BR-deficient mutants demonstrated that BR stimulates seed germination by reversing ABA-induced dormancy [9]. A recent study showed that 2 μM of exogenous BR reduced the inhibitory effect of high salt concentrations on seed germination and promoted early stages of seedling growth in *Brassica napus* [10]. Another study indicated that overexpression of the gene *AtDWF4*, essential for BR biosynthesis, was able to overcome ABA-induced inhibition of seed germination [11]. It has been proposed that exogenous BR can regulate other endogenous hormones, and the effect of BR on other plant hormones has been explored in several studies [12].

Plant miRNAs occupy only a small number of functional genes. Currently, 299 *A. thaliana* miRNAs are recorded in miRBASE (release 19) [13]. miRNAs bind to complementary sequences on target mRNAs and in plants mostly act to degrade them [14]. Complementary features of plant miRNAs target their mRNAs by an almost perfect match; most miRNA binding sites exist in coding exons [14,15]. Recently, miRNAs have been reported to be hypersensitive ubiquitous stress regulators: *i.e.*, they function to mediate expression of their target genes when unbalanced nutrient conditions are encountered [15]. miR399 and miR395 have been identified as being involved in sulfate- and phosphate-starvation responses [16,17].

While recent studies with a structural, genetic, molecular, transcriptomic and proteomic focus have helped elucidate the regulatory mechanisms of the BRs signaling pathway [18–21], the mechanisms of miRNA involvement with BRs are unknown. To gain insights into the mechanism of BR actions at the molecular level, we carried out global screening of miRNAs in *A. thaliana*, which responds rapidly to EBR treatment, successfully investigating potential targets of miRNA and their interaction in plant development.

## 2. Results and Discussion

### 2.1. EBR Regulates the Root Development of *Arabidopsis*

**Figure 1:**
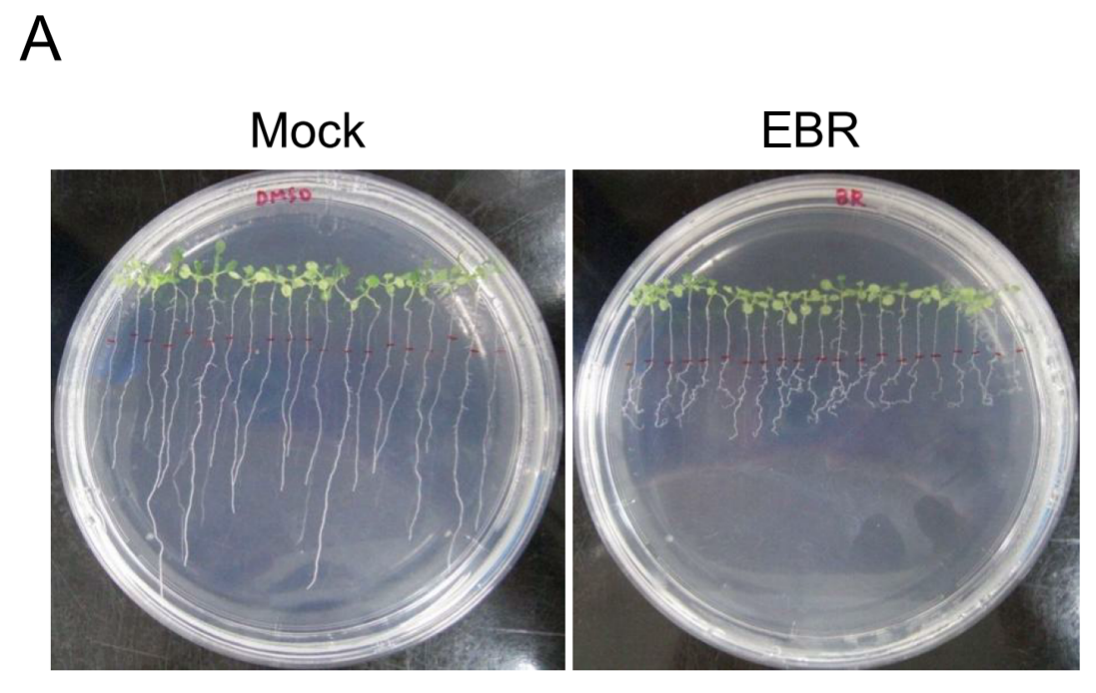

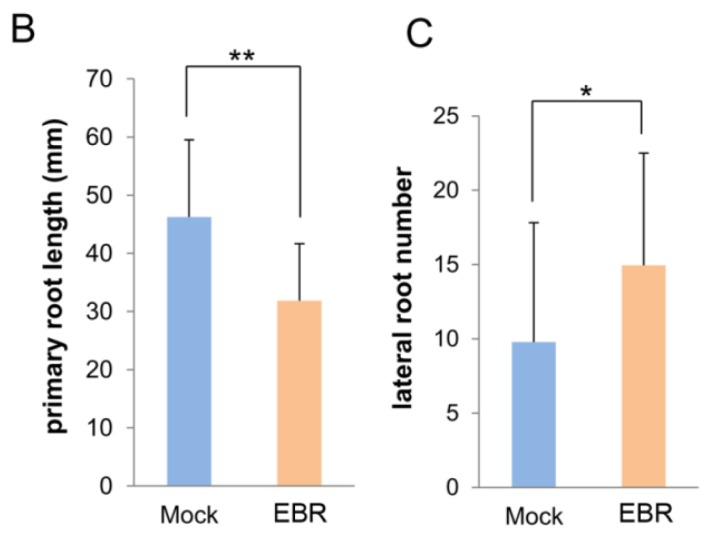
24-epibrassinolide (EBR) regulates root development. (**A**) The development of lateral roots was enhanced in EBR-treated seedlings. Each plate contained 10 nM EBR or mock solution (control). The red line represents initial length before treatment; (**B**) Differences in primary root length between day 5 and day 11. Primary root length was significantly shorter under EBR treatment; and (**C**) Number of lateral roots on day 11. Lateral root number was significantly increased in the EBR supplement plate. Representative data from three independent experiments are presented as mean ± SD. *p < 0.05, **p < 0.01 *vs.* control treatment values.

**Figure 2:**
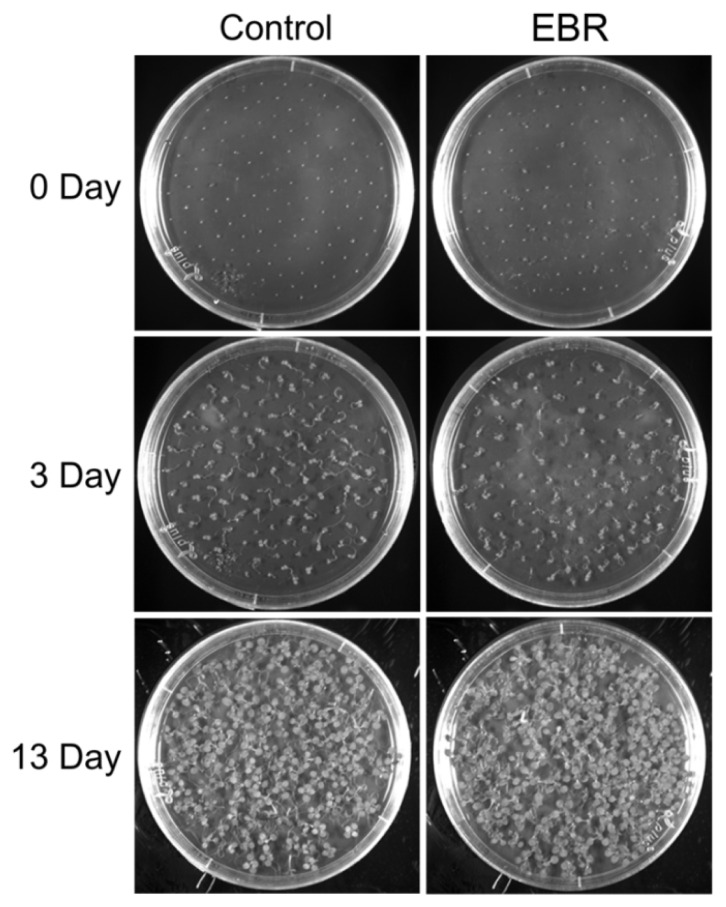
EBR has no effect on germination. There was no significant difference in germination rate between control and EBR-treated seeds; however roots were shorter and more strongly curved in EBR-supplemented plates. Germination was recorded on day 3 and day 13 after imbibition.

We monitored the morphology of EBR-treated seedlings for root length measurement and germination analysis. It has previously been shown that *Arabidopsis* with different levels of BRs display differences in root development [22,23]. Low concentrations (0.1 and 0.5 nM) of exogenous BRs promoted root elongation in wild-type strains and BR-deficient mutants [22]. In contrast, higher concentrations (1–100 nM) were inhibitory for primary root elongation, instead promoting lateral root formation [23,24]. In this study, we treated *Arabidopsis* with 10 nM EBR. Our results show that primary root length was significantly decreased (*p* < 0.01) and the number of lateral roots was significantly increased (*p* < 0.05) (Figure 1). Expression levels of *BRU6* and *SAURAC-1* genes in *Arabidopsis* were regulated by EBR stimulation (Figure S1), results that corroborate previous studies [21,24]. The development of germination was maintained after EBR treatment, but the root phenotype appeared obviously curved in our germination analysis (Figure 2). Based on these results, we confirm that the root development of *Arabidopsis* can be regulated by EBR at concentrations like those used in our treatments.

### 2.2. Identification of EBR-Regulated miRNAs in *Arabidopsis*

**Figure 3:**
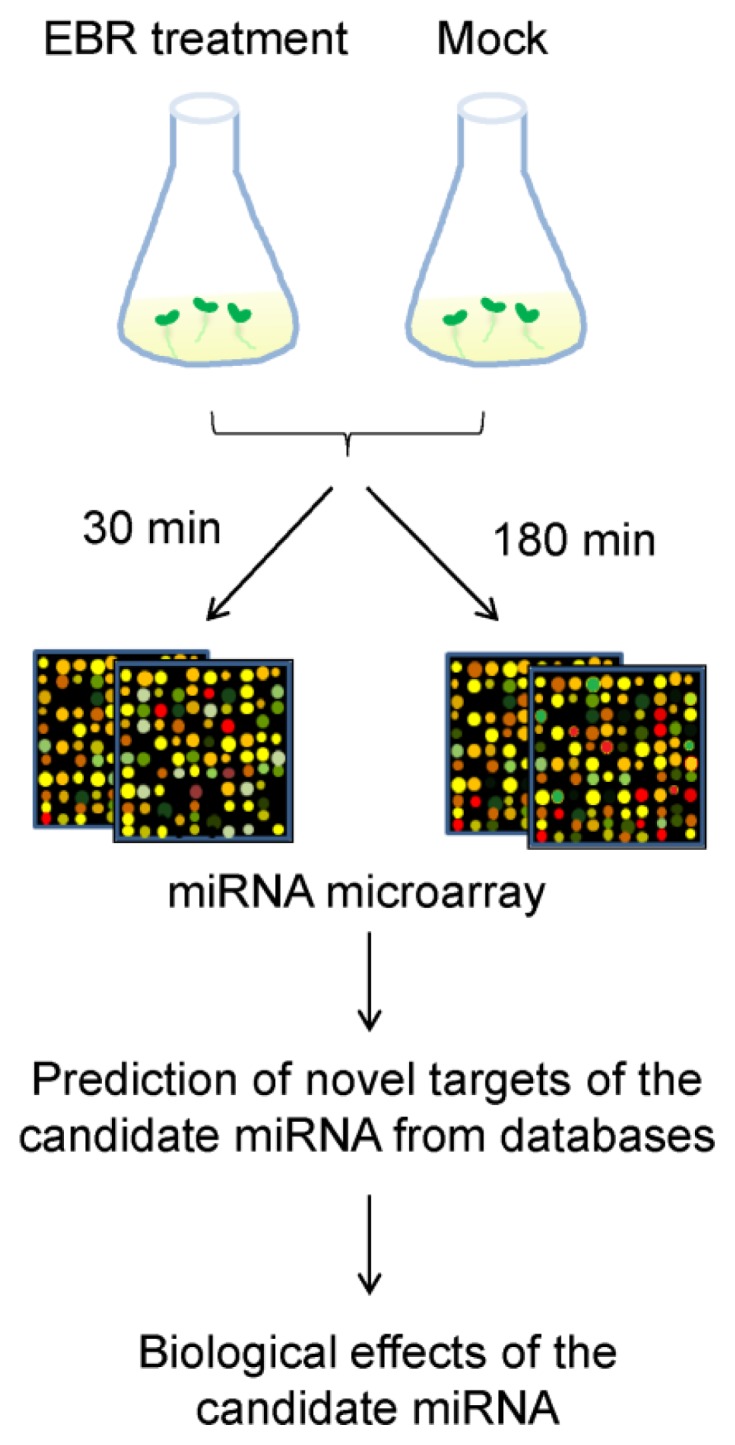
Schematic flowchart of experimental design. After *Arabidopsis* Col-0 seeds had grown in MS liquid medium for 7 days, seedlings were treated for 30 or 180 min with MS medium supplemented with EBR or mock solution (DMSO), followed by RNA extraction, labeling, and hybridization. Candidate miRNAs were predicted using miRU, WMD3, and psRNATarget databases. The roles of candidate miRNAs in EBR-treated seedlings were investigated by further experiments as described in the text.

To explore the role of miRNAs in BR-mediated pathways, we analyzed differences in miRNA profiles between control (mock solution) and EBR treatments from customized miRNA microarrays. Seeds were separately cultured under exogenous 10 nM EBR treatments for 30 (EBR30) or 180 (EBR180) minutes (Figure 3), and total RNA of all seedlings was extracted after seven days of growth. A scatter plot of probe intensities of duplicate microarrays shows no difference (*R*^2^ > 0.99) among the duplicates, therefore validating the consistency of our microarray experiments (Figure S2).

**Figure 4:**
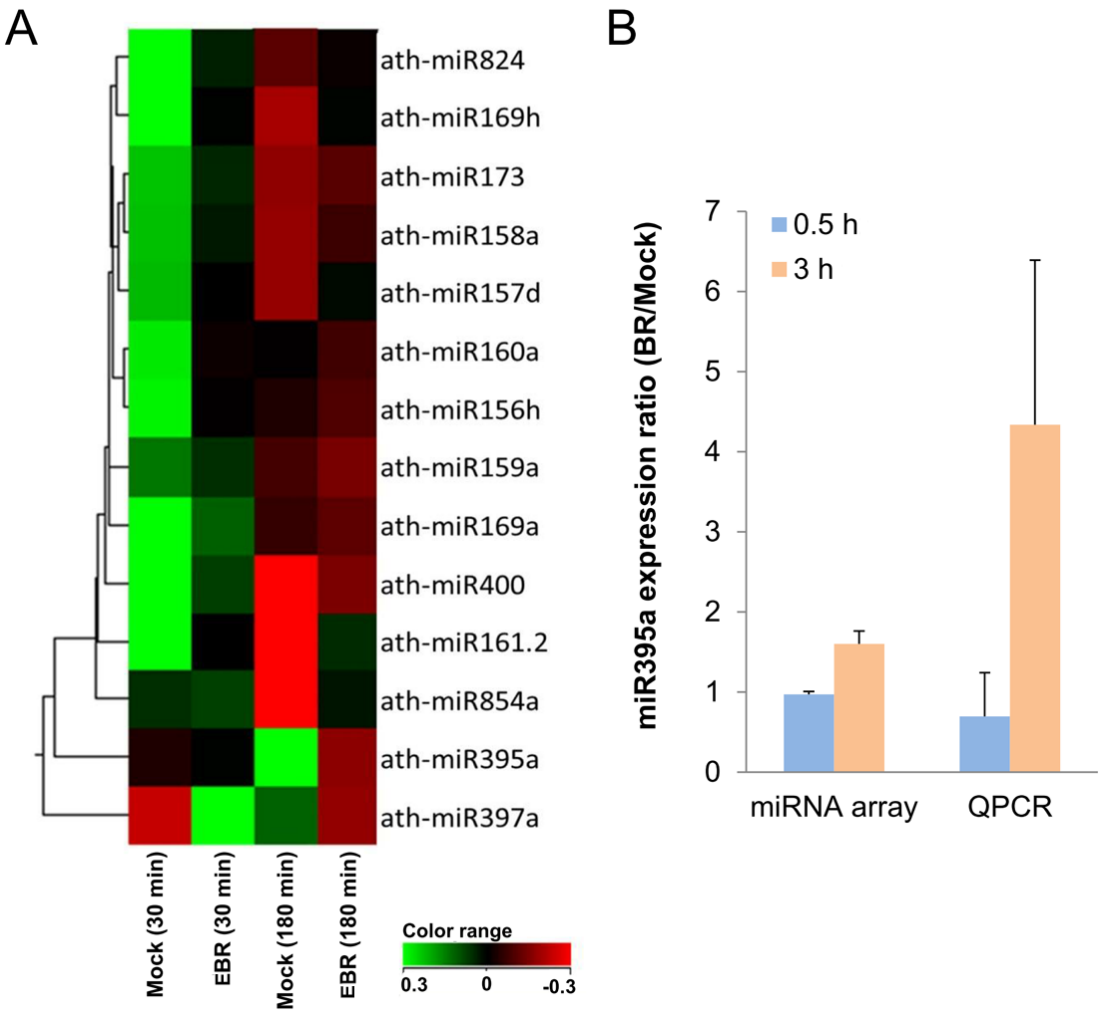
EBR up-regulates miR395a in miRNA microarray analysis. (**A**) Hierarchical clustering of selected miRNAs expression regulated by EBR. Seedlings were treated with EBR for 30 or 180 min. miRNA expression was assessed with miRNA microarrays. Fourteen miRNAs had significantly different expression levels after EBR treatment (*p* < 0.05) and were further analyzed by a hierarchical clustering algorithm; and (**B**) Fold changes of miR395a in miRNA microarrays and qPCR analysis. miR395a was up-regulated after EBR treatment for 180 min. snoR85 was used as an internal control for normalization.

The expressed fold changes of miRNAs from EBR-treated seedlings were normalized to a DMSO-treated control. Fourteen miRNAs with significantly different expression ratios (*p <* 0.05) (Table 1) from both EBR30 and EBR180 treatments were selected for hierarchical clustering (Figure 4A). Among these, 11 miRNAs were up-regulated and three down-regulated for EBR30, and six up-regulated and eight down-regulated for EBR180. Of these, miR395a exhibited the highest fold change (1.6-fold) from microarray data at EBR180. Similarly, EBR-treated seedlings showed a higher expression of miR395a (4.3-fold) than control seedlings in real-time PCR (qPCR) analysis (Figure 4B). These results validate that miR395a expression is up-regulated by EBR.

### 2.3. GUN5 Is a Novel Target of miR395a

**Figure 5:**
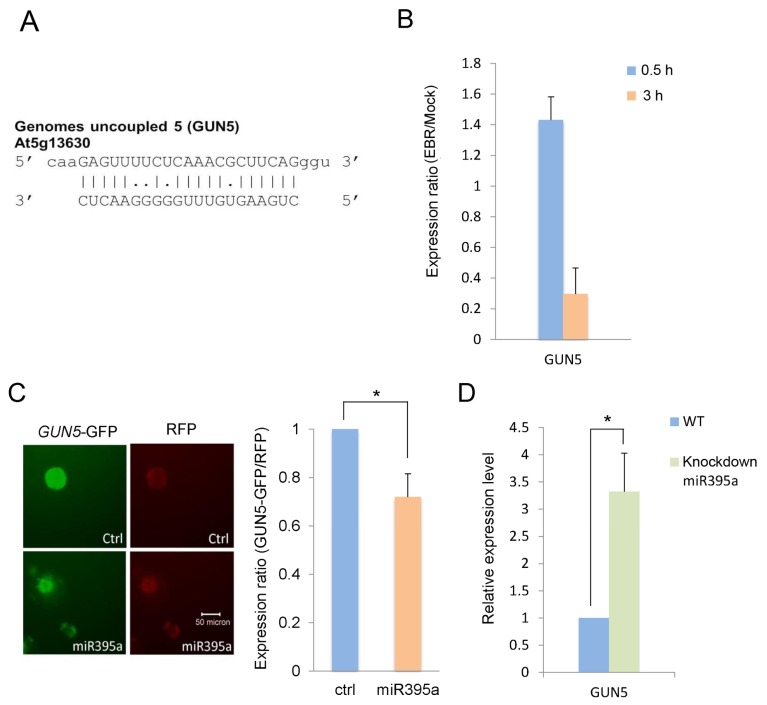
GUN5 is a target gene of miR395a. (**A**) The prediction was based on complementary base-pairing between miR395a and mRNA. Putative target genes for miR395a were predicted using web-based databases (miRU, psRNATarget, WMD3) and simultaneous comparisons to gene microarray data. The putative target genes of miR395a were down-regulated by EBR treatment; (**B**) Expression levels of GUN5 were analyzed by Q-PCR and normalized to 18S rRNA; (**C**) Fluorescence assay of miR395a and potential target GUN5 in the *Arabidopsis* PSB-D cell line. The fluorescent expression levels of GUN5 revealed significant down-regulation by miR395a; and (**D**) Gene expression of GUN5 measured by qPCR in miR395a knockout plants. GUN5 expression was significantly down-regulated by miR395a. *p < 0.05.

To investigate the role of miR395a in *Arabidopsis* development, we explored its potential targets by the bioinformatics approach of complementary base-pairing. We obtained potential target genes of miR395a from three databases: miRU [25] (Table S1), which integrates most known plant miRNAs and target genes and can be employed to search potential plant miRNA targets and target-sites within mismatch and miRNA conservation thresholds in target recognition; WMD3 [26] (Table S2), which uses principles of artificial miRNA design to mimic natural plant miRNAs; and psRNATarget [27] (Table S3), which provides scoring schemata and evaluates target-site accessibility in miRNA target recognition. The *Arabidopsis* TAIR9 cDNA library and default parameters were used in predicting target sequences from these databases. Among the candidate targets, *GUN5* (At5g13630), the CHLH subunit of Mg-chelatase, has been reported as possibly involved in the abscisic acid (ABA) pathway [28,29]. The interaction between GUN5 and miR395a is however still unknown. ABA, a plant hormone, mediates the development of plants by inhibiting seedling germination, maintaining primary root growth and reducing lateral root density [30–32]. We therefore decided to focus our investigation on whether *GUN5* is a target gene of miR395a; the target sequence of *GUN5* is shown in Figure 5A. We furthermore analyzed *GUN5* expressions in EBR- and DMSO-treated seedlings by qPCR. As shown in Figure 5B, these gene expressions were decreased in EBR-treated seedlings compared with DMSO-treated seedlings, indicating that *GUN5* might be associated with miR395a.

To further validate the interaction between miR395a and *GUN5* in *Arabidopsis*, we constructed pRTL2-miR395a and smGFP/pRTL2-*GUN5*. We co-transformed the plasmids into PSB-D cells and detected the fluorescence intensity of smGFP/pRTL2-*GUN5* and the internal control RFP. We also analyzed the levels of GFP-*GUN5* expression in transformed PSB-D cells. As shown in Figure 5C, lower *GUN5* expression was detected in PSB-D cells with miR395a overexpression than in PSB-D cells with control vectors. In plants with miR395a knockout, the gene expression of *GUN5* was also significantly increased (Figure 5D). These results suggest that *GUN5* is a target gene of miR395a.

### 2.4. Distribution of miR395a in Vascular Bundles, Leaf Veins and Roots of *Arabidopsis*

**Figure 6:**
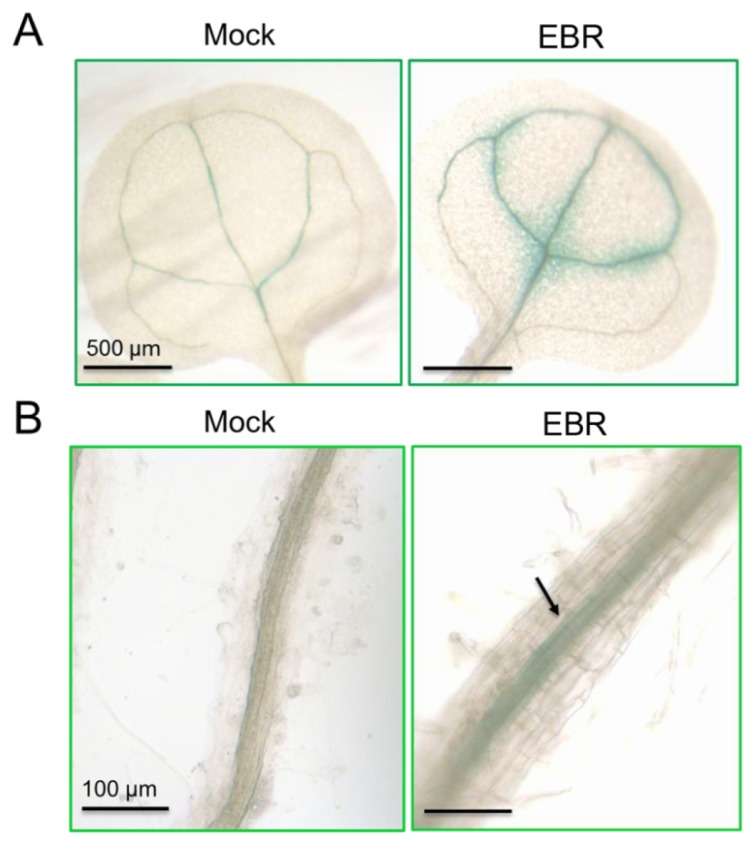
Histochemical GUS staining of miR395a expression in *A. thaliana*. Expression patterns of miR395a promoter::GUS plants in (**A**) leaf and (**B**) root tissue. After growing for 7 days, seedlings were grown under EBR treatment (10 nM EBR) or mock control for 3 h. The arrow indicates a high concentration of miR395a distributed in the vascular bundle compared with the mock treatment.

*GUN5* has been reported as being instrumental in leaf greening [28], and a decrease in chlorophyll accumulation has been found in *gun5* mutants [33]. BR is also a crucial factor in the regulation of chloroplast development, playing a role as a negative regulator [34]. Based on these similarities, we explored the role of miR395a in EBR-treated *Arabidopsis*, following *GUN5* suppression. To clarify the expression sites of miR395a, we used T2 seeds from miR395a promoter::GUS plants to examine the miR395a expression pattern under EBR treatment (expression levels of miR395a promoter::GUS were also found to be up-regulated under EBR treatment). In leaf and root development, miR395a specifically was concentrated in leaf veins of the cotyledon (Figure 6A) and in partial vascular bundles of roots (Figure 6B) and was also distributed in chloroplasts around leaf veins (Figure S3). Figure 6B also shows that root diameter under EBR treatment was larger under mock treatment, indicating that miR395a might regulate root development through EBR signaling.

**Figure 7:**
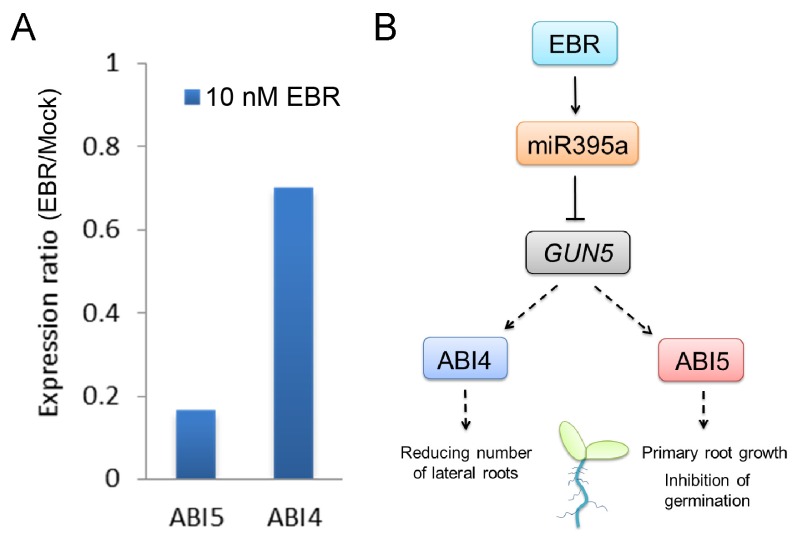
Expression of GUN5 downstream genes in the ABA pathway. (**A**) Relative expression ratio of GUN5 downstream genes; and (**B**) Diagram of GUN5-dependent ABA pathway.

A previous study has shown that the transcription factors *ABI4* and *ABI5* are positive regulators of ABA signaling and can be considered downstream genes of *GUN5* [19]. We found that these genes could be suppressed by EBR treatment (Figure 7A). It has also been reported that a mutation of *ABI4* can increase the number of lateral roots [35] and that *ABI5* activity inhibits seedling germination and promotes primary root growth [36,37]. These results lead us to propose that EBR may maintain seedling germination, inhibit primary root growth, and increase the number of lateral roots through regulation of miR395a effects on *ABI4* and *ABI5* via *GUN5* (Figure 7B).

### 2.5. Discussion

BRs can induce a wide range of physiological effects in cell elongation and division, photosynthesis, photomorphogenesis, flowering, senescence, seed germination, root development, male fertility, and abiotic and biotic stress resistance [8,19,38,39]. They are active at low concentrations throughout the plant kingdom and widely distributed in plants at varying levels of complexity [7,8]. Higher concentrations of BRs are seen primarily in young growing tissues rather than in mature tissues [7]. BR-insensitive mutants in *Arabidopsis* exhibit phenotypes such as dwarfism, dark-green leaves, reduced fertility, prolonged life span, and abnormal skotomorphogenesis [3,8,39].

miRNAs also provide examples of regulation at various stages of plant development. Some miRNAs, such as miR159 and miR160, play roles during early development stages including seed germination. During post-germination stages, miR156 and miR172 mediate the emergence of vegetative leaves, a stage of transition to autotrophic growth [40,41]. miRNA-mediated signaling is also involved in the development of various tissues; several miRNA families such as miR160, miR164, miR167, and miR390 have been demonstrated to be involved in root cap formation and lateral root development [42]. However, the relationship between BRs and miRNAs is unknown.

In the present study, we screened different miRNA expression profiles in *Arabidopsis* with 10 nM EBR for periods of 30 and 180 min. The results show that in both cases the expression of miR395a was significantly up-regulated by EBR (Figure 4). Recent studies have indicated that miR395a is up-regulated in roots and expressed in cortex, phloem companion cells and epidermis under low-sulfur conditions [43]. miR395a is mostly expressed in roots when playing a role in homeostasis regulation [43]. As discussed above, the morphology of BR-treated plants showed a decrease in taproot length and an increase in lateral root formation [23]. These effects might be caused by miR395a-involved mechanisms, and miR395a might be among the factors affecting root growth and development. miR395 and miR397 play roles in sulfate metabolism and copper homeostasis, respectively [43–45]. The function of these miRNAs lies mostly in adapting to unbalanced conditions, which implies that the experimental concentration of 10 nM EBR might have been in excess of physiological levels and affected the homeostasis of the seedlings.

In addition to miR395a, several significantly different expressions of miRNAs may have potential functions relevant to BR-treated seedlings:

(a) miR824 was down-regulated in BR-treated seedlings. It is involved in stomatal development by targeting *AGL16*, through which it causes a decrease in the number of stomata [46]. This suggests an increased stomata number in BR-treated plants [46,47]. Proper amounts and distributions of stomata are essential for successful gas exchange [46], and so an increase in the stomata number might therefore contribute to greater metabolic efficiency in plants.
(b) miR169a, which can regulate adaptive responses to nutrient deprivation [48], was also up-regulated in our miRNA profiles. This suggests that miR169a might have acted in this capacity of adaptation to environmental change when we supplied exogenous BR.
(c) miR160 mediates agravitropic roots with disorganized root caps as well as lateral root development, primary root growth, floral organs in carpels, and germination [40,42,49]. Our miRNA arrays indicated that the up-regulation of miR160a might have resulted in the expression of the phenotype observed in the present study. Since the lateral root formation caused by miR160 was similar to the morphology of BR-treated seedlings, we suspect miR160 might play an important role in lateral root development in BR-supplied plants.
(d) miR156 has been shown in recent studies to increase leaf initiation, phase change, floral induction, and phosphate homeostasis, to decrease apical dominance, and to delay flowering time [40,42,49]. As suspected, miR156h was up-regulated in the miRNA profiles, suggesting a crucial function in promoting growth and development.
(e) miR159 regulates germination, anthers, and flowering time by targeting the MYB transcription factor [49,50]. Overexpression of miR159 results in male sterility and delayed flowering time.

To further explore the role of miR395a, we predicted target genes of miR395a from several different databases and identified *GUN5* as a novel potential target of miR395a in *Arabidopsis.* We were able to show that the expression of *GUN5* was suppressible by miR395a (Figure 5C,D). Similar to the phenotype of the *gun4* mutant, the *gun5* mutant showed a decrease in chlorophyll accumulation, while the *gun4gun5* double mutant displayed the even more noticeable characteristic of albino leaves [28]. *GUN2/3/4/5* are also involved in communicating along plastid-to-nucleus retrograde signaling pathways with Mg-ProtoIX acting as a signaling molecule between chloroplast and nucleus [51]. In contrast, BR inhibits chloroplast development [34], and down-regulates *GUN5* expression (Figure 5B). After EBR treatment, miR395a was up-regulated and strongly expressed in cotyledon leaf veins and root vascular bundles (Figures 4B and 6). These results suggest that BR might enhance miR395a to suppress *GUN5* expression during plant development. However, the exact relationship between BR, miR395a and *GUN5* remains unknown. Recent studies have indicated that *ABI4* is a downstream regulator between chloroplast and nucleus that connects to ABA via retrograde signaling [52]. Hence, *GUN5* is likely to play a role in chlorophyll synthesis by connecting ABA to different pathways [33]. Additionally, we found that ABA regulatory genes were suppressed by EBR (Figure 7). These outcomes indicate that the interaction between miR395a and *GUN5* may regulate chlorophyll synthesis through the ABA signaling pathway.

## 3. Experimental Section

### 3.1. Plant Material and Growth Conditions

*A. thaliana* ecotype Columbia (Col-0) was used as plant material in this study. Before sowing, seeds were surface sterilized by rinsing them in 1% bleach (sodium hypochlorite) with 0.5% Tween 20 and vortexing for eight minutes, washed 5–6 times and then cold-treated for two days at 4 °C under dark conditions. Plants were sown in pots (containing 50% vermiculite and 50% soil mixture), medium, or agar plates and kept in a growth chamber operating at photoperiod conditions of 14 h light and 10 h darkness at 22 °C after stratification.

### 3.2. Germination Assay

Seeds were grown on half-strength Murashige and Skoog medium (1/2 MS medium; Duchefa Biochemie B.V., Haarlem, Netherlands) with 1.5% (*w*/*v*) sucrose (Sigma-Aldrich Co. LLC., Dorset, UK) and 0.8% (*w*/*v*) plant agar (Sigma-Aldrich, St. Louis, MO, USA) containing 10 nM 24-epibrassinolide (EBR, a highly active BR; Sigma-Aldrich) or mock solution (dimethyl sulfoxide, DMSO). Images were taken at zero, three, and thirteen days after sowing.

### 3.3. Root Growth Assay

For root elongation analysis, seedlings were grown vertically on 1/2 MS medium with 1.5% sucrose and 0.8% plant agar for five days after germination. Seedlings were then transferred to new plates containing MS medium supplemented with 10 nM EBR or mock solution for another six days, with images taken after five and 11 days. Differences in primary root length between the two images were measured, and number of lateral roots was calculated after 11 days, using ImageJ software [53].

### 3.4. MicroRNA Microarray Hybridization and Analysis

For the miRNA array experiments, seedlings were grown in 1/2 MS medium with 1.5% sucrose. After stratification, seeds were transferred into 50 mL flasks with 10 mL liquid medium and incubated for seven days at 50 rpm and 22 °C under continuous light conditions. Seedlings were then treated with medium containing 10 nM EBR or mock solution for 0.5 and 3 h, respectively. Total RNAs were extracted from complete frozen seedlings using TRIzol^®^ Reagent (Invitrogen, Carlsbad, CA, USA), and RNA purity was confirmed by spectrophotometry (A_260_/A_280_ ratio) and capillary electrophoresis (Agilent 2100 Bioanalyzer, Agilent Technologies, Palo Alto, CA, USA). Then, 100 ng total RNAs of each sample were prepared for labeling with Cyanine 3-pCp. RNA processing and hybridization were performed using miRBASE V14 arrays (Agilent Technologies, Palo Alto, CA, USA) according to the manufacturer’s protocol; this version contains 161 *Arabidopsis thaliana* miRNA genes. Each plex on these customized eight-plex microarrays contained duplicate or triplicate probes for each miRNA, with 20 replicates for each probe. Microarray analysis was carried out in GeneSpring GX version 11 (Agilent, city, state, country). The data (covering the four conditions) were classified into groups by the averages of duplicates, and the median of all samples was set as a baseline. Differences in miRNA expression were tested using a one-way ANOVA. miRNAs with significant differences (*p* < 0.05) between mock control and EBR-treated seedlings were selected for clustering and those with the highest fold change were subjected to further analysis. Array data were submitted to the GEO database (series record number GSE46377).

### 3.5. Real-Time RT-PCR

All cDNA synthesis was carried out on total RNAs using the RevertAid H Minus Reverse Transcriptase Kit (Fermentas, Maryland, NY, USA) according to the manufacturer’s instructions. Reactions for expression analysis of Col-0 genes treated with EBR or mock control were performed in triplicate and monitored using the iQ5 Real-time PCR Detection System (Bio-Rad, Philadelphia, PA, USA). Investigated genes and corresponding primers are listed in Table S5. Relative abundance of transcripts was normalized to the constitutive expression levels of 18S rRNA (At3g41768). For miRNA expression analysis, specific miRNAs were measured with TaqMan microRNA assays (Applied Biosystems, Foster City, CA, USA) according to the manufacturer’s instructions. All reactions were run in triplicate and snoR85 was used as the internal control for normalization.

### 3.6. Prediction of Novel miRNA Target Genes

We obtained *Arabidopsis* miRNA sequences from the miRBASE dataset [13]. The mature sequences of all miRNA genes were used in this study. For predictions of miRNA targets, the programs miRU [25], psRNATarget [27] and WMD3 [26] were employed. The *Arabidopsis thaliana* full genome (TAIR9) was selected in the psRNATarget and WMD3 databases (other parameters were left at default).

### 3.7. Vector Construction

The genomic DNA of *Arabidopsis* leaves was extracted using QuickExtract^™^ Plant DNA Extraction Solution (Epicentre, Madison, WI, USA), following the manufacturer’s instructions. Different vectors were used for specific purposes. For validating the interaction of miRNA and genes, vector pRTL2-mGFP (Biovector Co., LTD, Beijing, China) was used to construct miR395a and control. Vector pRTL2 was used to delete the mGFP gene from pRTL2-mGFP via the restriction enzymes *EcoR* I and *Xba* I of miR395a. Vector 326-RFP is an internal control for cell numbers in *Arabidopsis* cell lines. For cloning *GUN5* in translational fusion, the coding region of *GUN5* was inserted into smGFP/pRTL2 using the restriction site *Spe I*. Transcriptional fusions were created with GUN5 and smGFP for activity analysis in protoplast system. Protoplasts were prepared following the protocol of Miao and Jiang [54]. Vector pZP221 was used in transgenic plant construction for the miR395a-overexpressing line; the insertion containing miR395a with CaMV 35S promoter and terminator in the pRTL2-miR395a vector was cloned into the *Pst I* site of pZP221. The binary vector pBI101 with the reporter gene β-glucuronidase (GUS) was used for promoter activity analysis of the miR395a promoter line. Transcriptional fusions for analysis of promoter activity in plants were generated using the miR395a promoter with built-in cloning sites *Sal I* and *Xba I*, and GUS.

The miR395a knockout line was purchased from the *Arabidopsis* Information Resource (TAIR). Transformations were performed with the Gene Pulser Xcell^™^ Electroporation System (Bio-Rad, Richmond, CA, USA) at pulse settings of 130 V at 1000 μF.

### 3.8. Fluorescence Assay for Validating miR395a and GUN5

For fluorescence assays, 200 μL of transformed protoplast cells were transferred to black opaque 96-well microplates (Greiner Bio-One, Wemmel, Belgium) and immediately measured in a multimode microplate reader (FlexStation 3 microplate reader; MDS Analytical Technologies, Sunnyvale, CA, USA). Excitation and emission wavelengths were 488 and 508 nm for green light (smGFP) and 558 and 583 nm for red light (DsRed).

### 3.9. Detection of the Expression Pattern of miR395a in *Arabidopsis thaliana*

When *Arabidopsis* plants had grown for four to six weeks, the first bolt was cut to induce the emergence of further bolts. About one week after clipping, plants containing numerous unopened floral buds were immersed in a buffer of *Agrobacterium tumefaciens*. The buffer was prepared as follows: Transformed *A. tumefaciens* cells were grown at 28 °C and 180 rpm shaking in LB medium with the appropriate antibiotics. A 10-mL pre-culture was grown for two days and then transferred to the 200-mL main culture. This was incubated until an OD600 value of 0.8 was reached, and then was centrifuged at 4000× *g* for 15 min at 4 °C. The supernatant was discarded and sucrose and Silwet L-77 (Sigma, St. Louis, MO, USA) were added to the culture to obtain final concentrations of 5% and 0.05%, respectively.

Pots of plants were inverted and the inflorescence shoots dipped into suspension, then laid on a flat plastic surface and left covered and dark for the next 24 h, and afterwards returned to normal growing conditions. T1 plants were grown from selected transformants. The transgenic character of plants was confirmed by PCR and GUS staining.

When T2 trangenic lines were obtained, we used a GUS staining kit (GUSS; Sigma) to detect the expression pattern of miR395a. Seedlings were incubated at room temperature for 45 min with a fixation solution, which was then poured off. They were washed three times with wash solution for one minute, then left to incubate with staining solution for up to 24 h at 37 °C. Finally, the chlorophyll was removed by distaining the samples with ethanol. Tissues were stored in ethanol. Manufacturer’s instructions were followed in performing the assay.

### 3.10. Statistical Analysis

Data were represented as mean ± standard deviation (SD). Differences between independent groups were analyzed using a two-tailed Student’s *t-*test. MicroRNA microarrays for miRNA expression were analyzed using a one-way ANOVA (GeneSpring 7.3.1, Agilent Technologies, Palo Alto, CA, USA). A *p* value < 0.05 was taken to indicate statistical significance.

## 4. Conclusions

Our results show that miR395a was significantly up-regulated by EBR in *Arabidopsis*, was expressed more strongly in leaf veins and roots of EBR-treated miR395a promoter::GUS plants, and targeted GUN5 with the effect of suppressing its expression. EBR was able to suppress GUN5 downstream genes to regulate seedling germination and the formation of primary and lateral roots. These results suggest that the reduced amount of chlorophyll in leaf veins and root growth of *Arabidopsis* might be attributable to the interaction between miR395a and GUN5. This study provides new insights into the function of miRNAs that will be useful in further research into the roles miRNAs play in the molecular mechanisms of plant development.

## Supporting Information



## Figures and Tables

**Table 1:** EBR regulates the expression of several microRNAs (miRNAs).

microRNAs	Fold change (EBR/DMSO)	

	30 min	180 min
ath-miR824	1.27	0.93
ath-miR169h	1.37	0.86
ath-miR173	1.15	0.95
ath-miR158a	1.16	0.92
ath-miR157d	1.18	0.87
ath-miR160a	1.25	1.06
ath-miR156h	1.25	1.04
ath-miR159a	1.07	1.05
ath-miR169a	1.26	1.04
ath-miR400	1.21	0.89
ath-miR161.2	1.31	0.71
ath-miR854a	0.98	0.78
ath-miR395a	0.97	1.60
ath-miR397a	0.53	1.25
