# Supplementary material for: Identification of MicroRNA 395a in 24-Epibrassinolide-Regulated Root Growth of Arabidopsis thaliana Using MicroRNA Arrays
Source: Int J Mol Sci. 2013 Jul 9;14(7):14270–86. doi: 10.3390/ijms140714270 (PMC3742243; doi:10.3390/ijms140714270)
Supplement: Supplementary file 1 [file ijms-14-14270-s001.pdf]

Supporting Information

Figure S1. EBR-induced BRU6 and SAURAC-1 gene expression. Light-grown Col-0 seedlings were treated with 10 nM EBR and transcript abundance was analyzed by RT-PCR. Gene expression levels of (**A**) BRU6 and SAURAC-1; and (**B**) differential expression of each gene after EBR treatment for the indicated durations were compared to the DMSO-treated control and represented as expression ratios.

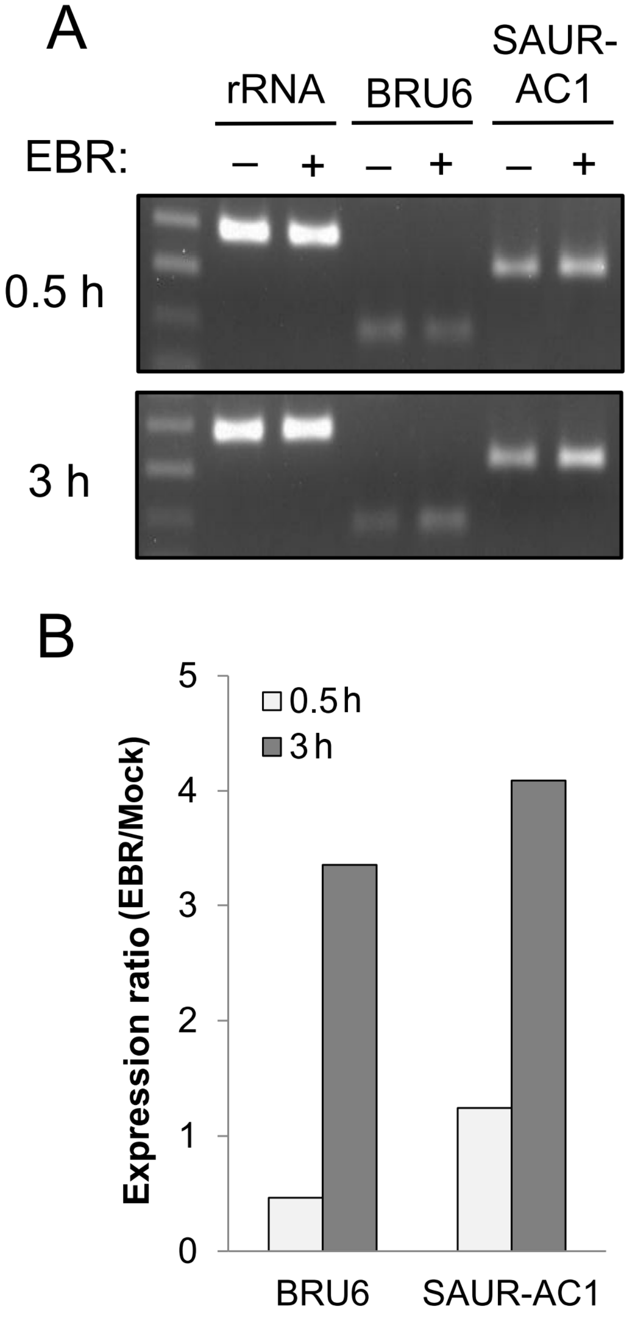

Figure S2. Signal scatter plot of sample duplicates. Seedlings were treated for (**A**) 30 or (**B**) 180 min with EBR or mock solution (DMSO). Each dot represents one miRNA. No differences between duplicates were found.

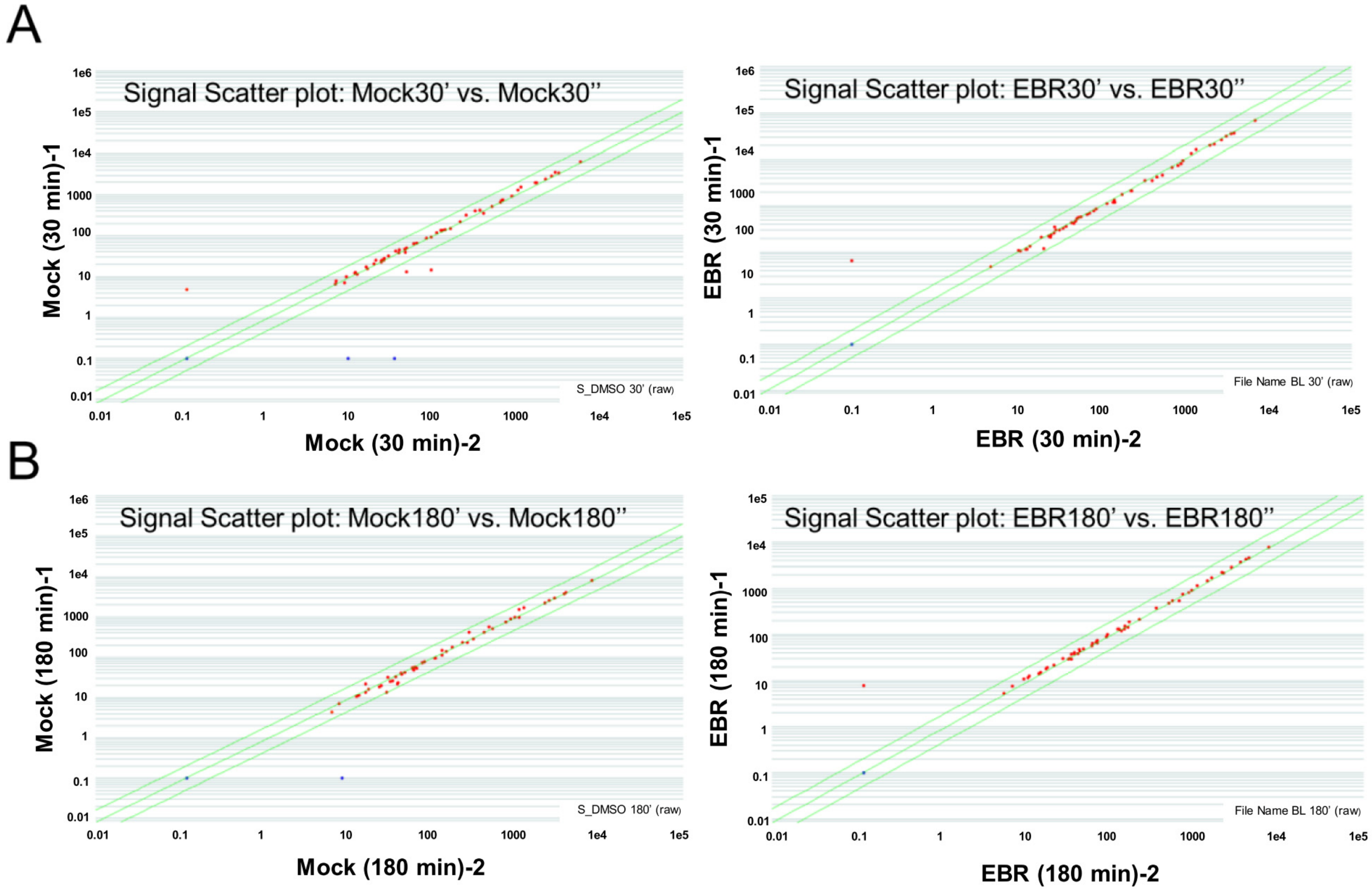

Figure S3. miR395a was expressed in chloroplasts around leaf veins. In miR395a promoter::GUS plants, leaf vein chloroplasts appearing as green spots show the expression pattern of miR395a.

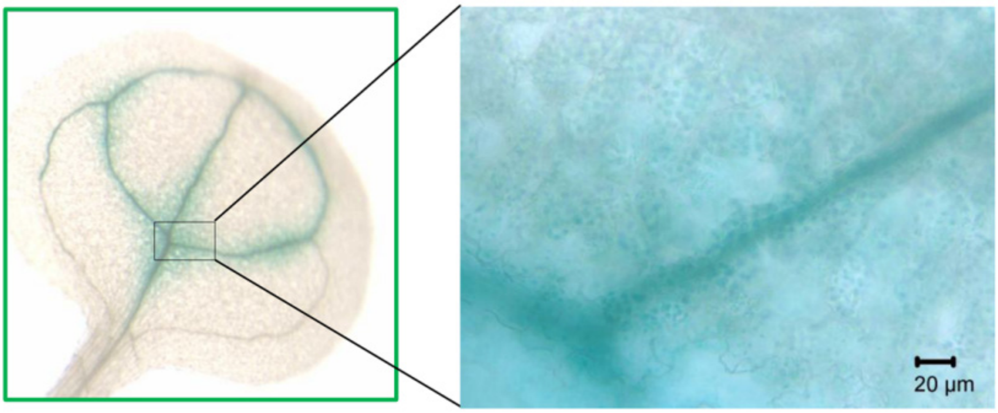

Table S1. Targets predicted by miRU database [1].

ID	Target site alignment	Site	Score	Mismatch
Query (3'-5')	cucaagggggguuugugaaguc			
At5g43780.1	gaguuccuccaaacacuucau	395–415	0.5	2
At2g28780.1	gagugccuccaaacacuucag	1119–1139	1.5	2
At5g10180.1	aaguucucccaaacacuucaa	125–145	1.5	3
At3g22890.1	gaguuccuccaaacucuucau	442–462	2	3
At4g14680.1	gaguuccuccaaacucuucau	377–397	2	3
At5g13630.1	gaguuuucucaaacgcuucag	3567–3587	2	4
At4g23840.1	gacuucccucaaaaacuucag	886–906	2.5	3
At2g19540.1	gaguuuccucacacacuuuau	280–300	2.5	5
At4g23990.1	caguuuccucaaacauuucaa	1030–1050	2.5	5
At2g21230.1	aaguuuccccaaccaauucag	1096–1116	3	4
At2g21230.2	aaguuuccccaaccaauucag	1096–1116	3	4
At3g13070.1	gaguucuucuaaacacuccaa	144–164	3	5
At3g54790.1	agguuccaccagagacuucag	3730–3750	3	5
At3g57300.1	aagugcucccagaaacuucag	1472–1492	3	5
At5g05170.1	caguuaccucaaaagcuucag	3001–3021	3	5

[1] http://bioinfo3.noble.org/miRNA/miRU.htm.

Table S2. Targets predicted by WMD3 database [2].

The potential targets of miR395a		
AT1G50930	AT2G28780	AT3G09220
AT3G22890	AT4G14680	AT4G23840
AT4G23990	AT5G13630	AT5G43780

[2] The website of WMD3: http://wmd3.weigelworld.org/cgi-bin/webapp.cgi.

Table S3. Targets predicted by psRNATarget database [3].

miRNA accession	Target accession	Expection	Alignment	Target description
submitted-miRNA	AT2G28780.1	1.5	miRNA 21 CUCAAGGGGGUUUGUGAAGUC 1<br>Target 1119 GAGUGCCUCCAAACACUUCAG 1139	Symbols: similar to unknown protein [Arabidopsis thaliana] (TAIR:AT3G09450.1), similar to P-type trefoil [Medicago truncatula] (GB:ABD32899.1); chr2:12347111-12350498 REVERSE
submitted-miRNA	AT5G10180.1	1.5	miRNA 21 UCAAGGGGGUUUGUGAAGUC 2<br>Target 126 AGUUCUCCCAAACACUUCAA 145	Symbols: SULTR2, AST68 (Sulfate transporter 2.1); chr5:3193145-3197121 FORWARD
submitted-miRNA	AT5G43780.1	1.5	miRNA 21 CUCAAGGGGGUUUGUGAAGUC 1<br>Target 395 GAGUUCCUCCAAACACUUCAU 415	Symbols: APS4; chr5:17606621-17608762 REVERSE
submitted-miRNA	AT1G50930.1	2	miRNA 21 CUCAAGGGGGUUUGUGAAGUC 1<br>Target 42 GAAUUCCCCCAAACACUUCAA 62	Symbols: similar to unknown protein [Arabidopsis thaliana] (TAIR:AT3G20557.1), similar to conserved hypothetical protein [Medicago truncatula] (GB:ABE91163.1), contains domain UNCHARACTERIZED (PTHR23140:SF6); contains domain UNCHARACTERIZED (PTHR23140); chr1:18880505-18881512 FORWARD
itted-miRNA	AT5G13630.1	2	miRNA 21 CUCAAGGGGGUUUGUGAAGUC 1<br>Target 3567 GAGUUUUCUCAAACGCUUCAG 3587	Symbols: CCH, CHLH, CCH1, GUN5 (GENOMES UNCOUPLED 5); chr5:4387337-4392230 REVERSE
submitted-miRNA	AT5G13630.2	2	miRNA 21 CUCAAGGGGGUUUGUGAAGUC 1<br>Target 3567 GAGUUUUCUCAAACGCUUCAG 3587	Symbols: CCH, CHLH, CCH1, GUN5 (GENOMES UNCOUPLED 5); chr5:4387337-4392230 REVERSE
submitted-miRNA	AT1G69792.1	2.5	miRNA 21 CUCAAGGGGGUUUGUGAAGUC 1<br>Target 11 GAGUUCUCCUGAACACUUCAU 31	Symbols: MIR395D; chr1:26273642-26273741 REVERSE

Table 1. *Cont.*

miRNA accession	Target accession	Expection	Alignment	Target description
submitted-miRNA	AT1G69797.1	2.5	miRNA 21 CUCAAGGGGGUUUGUGAAGUC 1<br>Target 11 GAGUUCCCUUAAACGCUUCAU 31	Symbols: MIR395F; chr1:26277521-26277632 FORWARD
submitted-miRNA	AT4G23990.1	2.5	miRNA 21 UCAAGGGGGUUUGUGAAGUC 2<br>Target 1064 AGUUUCCUCAAACAUUUCAA 1083	Symbols: CSLG3, ATCSLG3 (Cellulose synthase-like G3), transferase, transferring glycosyl groups; chr4:12456501-12460763 FORWARD
submitted-miRNA	AT1G26973.1	3	miRNA 21 CUCAAGGGGGUUUGUGAAGUC 1<br>Target 11 GAGUUCCUCUGAGCACUUCAU 31	Symbols: MIR395A; chr1:9363183-9363275 REVERSE
submitted-miRNA	AT1G69795.1	3	miRNA 21 CUCAAGGGGGUUUGUGAAGUC 1<br>Target 11 GAGUUCCUCUGAGCACUUCAU 31	Symbols: MIR395E; chr1:26276439-26276533 REVERSE
submitted-miRNA	AT2G17787.1	3	miRNA 21 UCAAGGGGGUUUGUGAAGUC 2<br>Target 1342 AGUUUUCCCAAGUAUUUUAG 1361	Symbols: similar to unknown protein [Arabidopsis thaliana] (TAIR:AT4G35940.1), similar to conserved hypothetical protein [Medicago truncatula] (GB:ABE79676.1); chr2:7738157-7740101 FORWARD
submitted-miRNA	AT2G20463.1	3	miRNA 21 CUCAAGGGGGUUUGUGAAGUC 1<br>Target 5 GAUUUUCCCCAAAAACUUUAG 25	Symbols: Encodes a defensin-like (DEFL) family protein; chr2:8830814-8831203 REVERSE
submitted-miRNA	AT3G22890.1	3	miRNA 21 CUCAAGGGGGUUUGUGAAGUC 1<br>Target 442 GAGUUCCUCCAAACUCUUCAU 462	Symbols: APS1 (ATP sulfurylase 3); chr3:8112730-8114997 FORWARD
submitted-miRNA	AT4G14680.1	3	miRNA 21 CUCAAGGGGGUUUGUGAAGUC 1<br>Target 414 GAGUUCCUCCAAACUCUUCAU 434	Symbols: APS3 (ATP sulfurylase 2), sulfate adenylyltransferase (ATP); chr4:8413283-8415386 REVERSE

[3] The website of psRNATarget: http://www.plantgrn.org/ psRNATarget/.

Table S4. Primer list designed for gene expression.

Gene ID	Primer name	F or R	Primer sequence (5'→3')
AT3G41768	A. tha: rRNA-1	forward	CGGCTACCACATCCAAGGAA
	A. tha: rRNA-2	reverse	GCTGGAATTACCGCGGCT
AT4G37390	A. tha: BRU6-1	forward	CGTATATCCAACGGCGATTGT
	A. tha: BRU6-2	reverse	CCAGCAGATGTTCCTGAGCTT
AT4G38850	A. tha: SAUR-AC1-1	forward	GAGGATTCATGGCGGTCTATG
	A. tha: SAUR-AC1-2	reverse	GTTAAGCCGCCCATTGGAT
AT5G43780	A.tha: APS4-1	forward	GCTTAGCATGGCACCAGGACTT
	A.tha: APS4-2	reverse	GCCAACCCACGCATCTTGGTT
AT5G13630	A.tha: GUN5-1	forward	GGCTGGACGCAAGAACAAAG
	A.tha: GUN5-2	reverse	GCACTCCATCCCACAGTGTTG
AT2G21230	A.tha: F7O24.5-1	forward	AACTCCGCGATGCTCTGTCAGAGA
	A.tha: F7O24.5-2	reverse	GTTCGGCTCCCCTATCACCAGTTT
AT4G33430	A.tha: BAK1-1	forward	CTGGACAGCTCGTAATGCAA
	A.tha: BAK1-2	reverse	GGCCGAGAGTTGATGGAATA
AT1G19350	A.tha: BES1-1	forward	CTACCCGTTTTATGCGGTGT
	A.tha: BES1-2	reverse	TTGCTGCTGTGTTTGGAGAC
AT1G75080	A.tha: BZR1-1	forward	TCATCATTCCCGAGTCCTTC
	A.tha: BZR1-2	reverse	AATGGCCATGGATTGCTTAG
AT4G39400	A.tha: BRI1-1	forward	GCAACTGTTCTGAGCTGGTTTCGC
	A.tha: BRI1-2	reverse	TGAGCTCCTGAGGGATCTCTCCT

© 2013 by the authors; licensee MDPI, Basel, Switzerland. This article is an open access article distributed under the terms and conditions of the Creative Commons Attribution license (http://creativecommons.org/licenses/by/3.0/).
